# Practical Notes on Diagnosis and Treatment

**Published:** 1906-11-24

**Authors:** 


### PRACTICAL NOTES ON DIAGNOSIS AND TREATMENT.

### Ice-bag in Infantile Paralysis.

In infantile paralysis, if seen within 48 hours of the onset, an ice-bag, if applied over the affected portion of the spine, may be expected to render good service.—*Dr. Lees.*

### The Ice-bag in Appendicitis.

The local application of an ice-bag is often of great benefit in the less acute inflammations of the vermiform appendix (perityphlitis). It often produces very rapid relief of pain and diminution of the swelling. Anyone who watches the effect of the ice-bag on this purely local inflammation will be prepared to accept its local influence in pericarditis and in pneumonia.—*Dr. Lees.*

### Acne Rosacea.

Ichthyol given internally acts well in this disease; it often brings about an improvement in the course of a few days. To begin with, five grains should be taken on an empty stomach night and morning. If necessary, the dose is gradually increased until the desired object is attained. Some patients react to a small dose, while others need larger quantities.—*Mr. Malcolm Morris.*

### Periostitis.

When called to see a case of febrile disturbance of a doubtful nature either in infants, children, or adults, but especially during the age of growth, carefully examine the limbs, and particularly the long bones, for swellings. I have frequently been called in to see young people supposed to be suffering from rheumatism, and have on examination of the limbs ascertained the case to be one of ostitis or periostitis. *Mr. Thos. Bryant.*

### Turpentine in Gall-stone Colic.

Turpentine possesses qualities that are valuable in the treatment of cases of gall-stone. It stimulates the expulsive action of the gall-bladder and ducts. It also increases the secretion of bile and prevents decomposition of that fluid. When given for a length of time it arrests the formation of "recurrent" gall-stones. Five minims thrice daily may be ordered either with mucilage or in a capsule.—*Dr. Ralfe.*

### Iritis.

For the diagnosis of iritis observe, first, the colour of the iris, next its lustre, and then the size, shape, and reaction of the pupil. And to appreciate changes in one or all of these directions, carefully compare the affected with the sound eye. As treatment in a recent case, a drop of atropine solution should be applied to the inner aspect of the lower lid every quarter of an hour for two hours, and then once every three or four hours until the pupil is dilated. Leeches, hot fomentations, and free purgation will aid the action of the mydriatic.

### Erysipelas.

Equal parts of ichthyol and lanoline make a useful application in erysipelas. This ointment should be smeared freely over the affected area and covered with salicylic gauze.

### Ischuria in Hysteria.

Ischuria, at least in a moderate degree, is more common in hysteria than is usually thought to be the case. Sometimes it exists in a very extreme form. I have seen a patient who only passed 16 oz. of urine in the course of a week, and that, too, apart from vomiting.—*Dr. Robert Saundby.*

### Lithiasis in Children.

Frequency of micturition, with pain in the neck of the bladder and urethra, and hæmaturia, occurring in children, do not necessarily mean a stone in the bladder. These symptoms may be due to crystals of uric acid or oxalate of calcium in the urine.—*Mr. Jno. H. Morgan.*

### Vaginismus.

In cases associated with such conditions as piles, caruncle, fissure, ulceration, etc., the treatment is to get rid of these conditions. In primary cases—that is, cases not dependent on any appreciable local cause—treatment is often difficult. It may be necessary to ascertain the sexual condition of the husband, or to separate husband and wife for some considerable time. Dilatation under ether with persistent use of a glass dilator succeeds in some cases. In others, cocaine applied locally immediately prior to intercourse may be successful.

### Drugs in Bronchiectasis.

Give quinine in full doses, say three grains thrice daily; also gum resins such as copaiba, chian turpentine, tar, Friar's balsam, and turpentine. These help to diminish the fœtor and quantity of the expectoration. Tincture of musk (grs. ij in ʒj) in drachm doses is also of value. As an inhalation the following may be recommended:—Carbolic acid, creasote, tincture of iodine, ether, of each 2 drachms, rectified spirit 4 oz. Half a drachm may be placed on lint and inhaled from a Coghill's inhaler several times a day.—*Sir Dyce Duckworth.*

### Atropine in Iritis.

If adhesions in iritis are many and strong, many hours, and even days, may elapse before appreciable dilatation of the pupil is effected by atropine. In many cases the adhesions only give way during the processes of resolution and repair, but they will not give way even then unless the iris be kept under the influence of atropine. As to the length of time during which atropine should be continued, the rule is to use it until all the external redness has disappeared, and for a further period of 10 to 14 days. *Sir John Tweedy.*

